# The saturation effect of blended pedagogy on international students’ acculturation in Confucian classrooms

**DOI:** 10.3389/fpsyg.2026.1803766

**Published:** 2026-06-12

**Authors:** Hong Zhang, Wen Li, Jiaying Qian, Qunsen Dai, Chen Wang, Chao Zhou

**Affiliations:** 1 Shaoxing University, Shaoxing, China; 2 Shaoxing Institute of Technology, Shaoxing, China; 3 Department of Psychology, Shaoxing University, Shaoxing, China; 4 Center for Brain, Mind and Education, Shaoxing University, Shaoxing, China; 5 Faculty of Continuing Education, Zhejiang University of Technology, Hangzhou, China

**Keywords:** blended pedagogy, cognitive load, cultural education, international students, saturation effect, ward’s ABC framework

## Abstract

Blended pedagogy, combining lectures and practice, is widely adopted in cultural education, with the expectation that combining multiple pedagogies leads to better cross-cultural adaptation. However, whether this assumption holds when pedagogies are combined at high frequency in the unique, high-context setting of Confucian classrooms remains unclear. Guided by Ward’s ABC (Affective, Behavioral, and Cognitive) framework, this study has investigated whether a saturation effect emerges when lecture-based and practice-based pedagogies are frequently combined. Survey data from 386 international students (30.3% female; *M_age_* = 23.32, *SD* = 7.78) showed that while practice-based pedagogy enhanced all ABC domains (*β* = 0.14–0.21, *p* < 0.01) and lecture-based pedagogy solely benefited behavioral skills (*β* = 0.12, *p* < 0.05), their high-frequency combination significantly reduced students’ behavioral adaptation and cultural identity (*β* = −0.13 to −0.14, *p* < 0.05). This finding can be interpreted through the lens of Cognitive Load Theory, suggesting that the need to understand high-context social cues while engaging with diverse teaching styles may overwhelm students’ working memory, hindering deeper adaptation. These findings challenge the “more-is-better” assumption and offer practical insights for curriculum design in cultural education.

## Introduction

Blended pedagogy, defined in the present study as a combination of lecture-based instruction and practice-based activities, is widely used in cross-cultural educational contexts. Its use is often guided by the implicit “more-is-better” assumption that providing diverse and frequent instruction leads to better cross-cultural adaptation outcomes. This assumption is particularly relevant for international students, who must adapt to new cultural and academic environments. However, this assumption may not hold universally, as suggested by Cognitive Load Theory (Sweller, 1988). When facing high cognitive demands in navigating unfamiliar social and academic environments, international students may not benefit from diverse and frequent instruction in their cultural adaptation.

According to the theory of cognitive load, learners have limited working memory capacity (Sweller, 1988). When instructional designs require an individual to process multiple information sources or switch rapidly to different task formats, the resulting cognitive load can impair rather than facilitate learning. This principle of limited working memory capacity has been extensively researched in general educational contexts (Ginns and Leppink, 2019), but its implications for cross-cultural education remain underexplored. International students face a unique dual demand to master new academic content while dealing with unfamiliar social norms and communication styles (Kim, 2001). Recent research has highlighted the acculturative challenges faced by international students in China (Yuan et al., 2024), underscoring the need for effective cultural education to facilitate their adaptation.

Previous research on cross-cultural education has shown that structured learning experiences can enhance cultural knowledge and skills (Wang et al., 2021a; Wang et al., 2022). Research has also shown that engagement with host communities can strengthen cultural identity (Esses et al., 2021), further reinforcing the “more-is-better” logic. However, it remains unclear whether it applies in classrooms influenced by Confucian heritage, which are characterized by high-context communication and high power distance (Hofstede and Bond, 1988; Li, 2012). These features persist in contemporary Chinese classrooms and are likely to characterize the cultural education courses attended by international students in China, as these courses are taught by Chinese instructors within the Chinese educational system. In these settings, students already dedicate significant cognitive resources to decoding implicit social and instructional cues. Adding the demand of frequently switching between passive (lecture) and active (practice) pedagogical modes may therefore push learners beyond their cognitive processing capacity. Consequently, this may generate a saturation effect, where additional instructional variety yields diminishing or even negative returns rather than enhanced adaptation.

China provides a strategic context to examine this possibility. With over 125,000 international graduates in 2022 alone (Ministry of Education of the People's Republic of China (MEPRC), 2024), the country has become a leading destination for students from Asia and Africa (UNESCO, 2023; Jianni, 2017; Wei, 2013). Additionally, since 2018, the Chinese government has mandated that universities offer formal cultural education courses to support the acculturation of this growing population (Ministry of Education of the People's Republic of China (MEPRC), 2018). More importantly, these courses typically employ blended pedagogy, combining lecture-based instruction on Chinese society, history, and values with practice-based activities such as community service. This policy-driven implementation provides an ideal setting to test whether the saturation effect occurs in a real-world Confucian classroom.

Existing research on international students in China has documented cross-cultural adaptation patterns (Bai et al., 2023) and highlighted the importance of contextual factors in acculturation (Zhang and Zeng, 2023). However, the role of pedagogical intensity in shaping these outcomes remains underexplored. To systematically assess adaptation outcomes of international students, the present study adopts Ward’s ABC framework, which has been widely applied in multiple research settings (Ward and Geeraert, 2016). According to this framework, three core domains have been distinguished as Affective (A, e.g., cultural identity), Behavioral (B, e.g., intercultural skills), and Cognitive (C, e.g., knowledge of norms) (Ward and Geeraert, 2016). This framework is suitable for the present study because it allows for domain-specific examination of how different pedagogies may affect different aspects of acculturation. In educational settings, lecture-based instruction is often assumed to target cognitive outcomes, whereas practice-based activities are assumed to target behavioral and affective development (e.g., Silva et al., 2022; Simpson, 2017; Wong and Driscoll, 2008). However, direct empirical evidence linking specific pedagogies to distinct ABC domains remains limited. Furthermore, a critical gap is not whether blended pedagogy works in general, but whether its high-frequency combination, intensive use of both lectures and practice, may become ineffective or even counterproductive in high-context Confucian classrooms.

To address this gap, the present study investigates two specific questions. First, what are the unique contributions of lecture-based and practice-based pedagogies to international students’ acculturation across affective, behavioral, and cognitive domains? Second, and more critically, does their high-frequency combination produce a negative interaction (i.e., a saturation effect)? By answering these questions, we aim to test the boundary conditions of the ABC framework in a high-context educational setting and to offer a cognitive-psychological explanation of when and why blended pedagogy may fail, thereby providing evidence-based insights for designing culturally responsive cross-cultural education programs beyond any single national context.

## Methodology

### Participants

A sample of 386 international students (30.3% female; *M_age_* = 23.32, *SD* = 7.78) from 67 countries, recruited from nine Chinese universities, participated in the survey. The sample reflected China’s international student demographics, with the majority originating from Asia (e.g., Pakistan, Bangladesh) and Africa (e.g., Nigeria, Morocco), consistent with national enrollment patterns (Ministry of Education of the People's Republic of China (MEPRC), 2024).

### Measurement

#### Demographic form

A self-developed demographic form was used to collect demographic data on age, gender, degree level, religion, ethnicity, duration of stay in China, and cultural distance. Notably, the frequency of lecture and practice-based pedagogies was assessed using two self-report items referring specifically to the cultural education courses participants attended. Items are “During your cultural education courses, did you frequently experience lecture-based teaching on Chinese culture (e.g., history, values, and social norms)?” and “During your cultural education courses, did you frequently experience practice-based activities such as community service or cultural immersion?” (Response options: yes/no). The term “frequently” was left to students’ own subjective interpretation without a standardized time-based definition (e.g., specific hours per week). Aligning with prior acculturation studies emphasizing subjective experience (e.g., Zhang and Ren, 2024; Zhang and Zeng, 2023), this approach captures perceived instructional intensity, which prior acculturation research has emphasized as more relevant to adaptation outcomes than objective counts (Ward and Geeraert, 2016). The survey instructions distinguished between in-class lectures (lecture-based) and out-of-class activities (practice-based), but did not further differentiate specific types of practice (e.g., community service vs. cultural immersion). The questions referred to the entire duration of the cultural education courses rather than a specific time window.

#### Subscales of the cultural intelligence scale

The cognitive and behavioral domains of acculturation were assessed using the Cultural Intelligence Scale’s cognitive (6 items) and behavioral (5 items) subscales, respectively, showing good reliability and validity (Ang et al., 2007). Responses are recorded on a 7-point Likert scale (1 = strongly disagree, 7 = strongly agree). An example item on the cognitive subscale is “I know the rules for expressing non-verbal behaviors in other cultures”. An example item on the behavioral subscale is “I change my nonverbal behavior when a cross-cultural situation requires it.” In the present study, Cronbach’s *α* values for the cognitive and behavioral subscales are 0.92 and 0.90, respectively.

#### Chinese cultural identity questionnaire

The affective aspect was measured using the Chinese Cultural Identity Questionnaire, which has demonstrated good reliability and validity in previous studies with international students in China (Zhang et al., 2011). The questionnaire comprises 22 items, each rated on a 5-point scale from 1 (strongly disagree) to 5 (strongly agree). Example items are “I like Chinese traditional culture” and “I discuss or participate in Chinese popular things”. In the present study, the Cronbach’s α value exceeds 0.90.

### Procedure

Before the study was conducted, the research team received ethical approval from the University Human Research Ethics Committee of Shaoxing University. Participants were recruited from nine Chinese universities. Emails/messages were sent to international students with assistance from the Offices of International Student Management at these Chinese universities. They were invited to complete a 15-min online survey on the Wenjuanxing (SoJump) platform. On the first page of the questionnaires, an informed consent form was presented, and participants indicated their consent by proceeding to the survey. In the survey, a battery of questionnaires was used in both Chinese and English to ensure readability.

### Statistical analyses

Data analyses were conducted using SPSS v22.0. Descriptive analyses were conducted of the pedagogies of cultural education and acculturation outcomes (i.e., ABC domains). Correlations among ABC were also presented. Hierarchical regression analyses were used to examine the association between cultural education and ABC, controlling for gender, religion, degree level, cultural distance, and duration of stay in China.

The cultural distance measure, along with several other exploratory items not included in the current study, was located on the final page of the survey. Consequently, data for this measure were missing for 71 participants (18.4%) due to incomplete survey submission. Missing data were handled using the Expectation–Maximization (EM) algorithm in SPSS. The imputation model included all variables from the main analyses (gender, religion, degree level, Duration of stay, lecture frequency, practice frequency, and the three acculturation outcomes). Following imputation, all analyses were conducted on the full sample of 386 participants. As a sensitivity check, all analyses were re-run using complete-case data (*N* = 315).

## Results

### Descriptive statistics

Table 1 displays detailed demographic information of the sample. There were more female participants than male participants, consistent with the gender distribution of international students pursuing higher education in China. These students had various majors from multiple Faculties. The majority of them had scholarships and religious beliefs. Approximately half of the students passed the low-level Chinese Proficiency Test (HSK level 3 or below), and another quarter had not completed the test. The sample was distributed equally across degrees. More than half of the participants had studied in China for up to 2 years.

**Table 1:** Demographic characteristics of the sample (*N* = 386).

Variable	*n*	Percentage
Gender		
Male	269	69.7%
Female	117	30.3%
Religion		
Yes	346	89.6%
None	40	10.4%
Degree level		
Bachelor	119	30.8%
Master	107	27.7%
Doctorate	147	38.1%
Others	13	3.4%
Duration of stay in China		
< 1 year	144	37.3%
1-2 years	60	15.5%
2-3 years	35	9.1%
3-4 years	44	11.4%
> 4 years	103	26.7%

Descriptive statistics and correlations of the learning outcomes are shown in Table 2. On average, these participants’ cultural knowledge and skills were at a medium-to-high level, and their cultural identity was relatively strong. All three aspects of learning outcomes were significantly correlated (*p* < 0.001).

**Table 2:** Descriptive statistics and correlations of the learning outcome (*N* = 386).

Variable	M	SD	Range	1	2
1. Cultural knowledge (C)	4.86	1.35	1–7	—	
2. Cultural skills (B)	4.93	1.36	1–7	0.81^***^	—
3. Cultural identity (A)	4.28	0.70	1–5	0.63^***^	0.54^***^

***p < 0.001.

Regarding the reported frequency of pedagogical exposure, lecture-based instruction was used more frequently (327 of 386 students, 84.7%) than practice-based activities (176 of 386 students, 45.6%). Among the participants, 150 students (38.9%) reported experiencing both pedagogies at high frequency, constituting the key group for examining the hypothesized interaction effect. Compared with practice, lectures have been used more frequently, reflecting the prevailing educational conditions in China.

### Regression analyses

Regression analyses were conducted for cultural knowledge, skills, and identity. Predictors (i.e., high-frequency use of lecture and practice-based pedagogies) were mean-centered prior to analysis, and multicollinearity was not present (VIFs < 1.1). Demographic variables, including gender, religion, degree level, duration of stay in China, and cultural distance, were initially included as control variables in the regression. Two teaching pedagogies and their interaction were entered in the following steps. As shown in Table 3, after controlling for the demographic variables, cultural knowledge, skills, and identity were significantly positively predicted by cultural education. Specifically, high-frequency use of the lecture alone contributed only to skills (*β* = 0.12, *p* < 0.05), whereas practice training enhanced all ABC domains (*β* = 0.14–0.21, *p* < 0.01). However, a key finding was that the interaction term between the high-frequency use of lecture-based and practice-based pedagogies had a significant negative impact on cultural skills (B) (*β* = −0.13, *p* < 0.05) and cultural identity (A) (*β* = −0.14, *p* < 0.05). This indicates that when both pedagogies are used intensively in combination, they do not exhibit the expected benefits but instead impede students’ behavioral and affective adaptation.

**Table 3:** Hierarchical regression analysis of perceived pedagogy frequency effects on ABC acculturation (*N* = 386).

Predictor variables	Cultural knowledge (C)				Cultural skills (B)				Cultural identity (A)			
	*B* [95% CI]	*β*	*t*	*sr^2^*	*B* [95% CI]	*β*	*t*	*sr^2^*	*B* [95% CI]	*β*	*t*	*sr^2^*
Demographics												
Gender	−0.18 [−0.47, 0.11]	−0.06	−1.20	0.004	−0.04 [−0.33, 0.26]	−0.01	0.18	<0.001	−0.20 [−0.35, −0.05]	−0.13	−2.60^*^	0.018
Degree level	0.02 [−0.13, 0.17]	0.01	0.26	<0.001	<0.01 [−0.15, 0.16]	<0.01	−0.57	<0.001	0.01 [−0.07, 0.09]	0.02	0.33	<0.001
Religion	0.13 [−0.31, 0.57]	0.03	0.59	0.001	0.04 [−0.40, 0.49]	0.01	−0.31	<0.001	<0.01 [−0.23, 0.23]	<0.01	−0.02	<0.001
Duration of stay	0.11 [0.03, 0.19]	0.14	2.67^**^	0.019	0.06 [−0.22, 0.14]	0.07	0.62	0.005	0.05 [0.01, 0.10]	0.13	2.51^*^	0.016
Cultural distance	0.03 [−0.08, 0.14]	0.03	0.53	0.001	0.05 [−0.06, 0.16]	0.05	0.73	0.002	0.03 [−0.03, 0.09]	0.05	1.00	0.003
*ΔR^2^*			0.022				0.007				0.035^*^	
Cultural education												
Lecture-based	0.08 [−0.06, 0.21]	0.06	1.08	0.003	0.17 [0.03, 0.30]	0.12	2.20^*^	0.016	0.04 [−0.03, 0.11]	0.06	1.23	0.004
Practice-based	0.28 [0.15, 0.41]	0.21	4.12^***^	0.043	0.20 [0.06, 0.33]	0.14	3.19^**^	0.021	0.10 [0.03, 0.17]	0.14	2.86^**^	0.021
*ΔR^2^*			0.045^***^				0.036^**^				0.024^**^	
Interaction												
Lecture × Practice	−0.13 [−0.26, 0.002]	−0.10	−1.94	0.010	−0.18 [−0.32, −0.05]	−0.13	−2.28^*^	0.019	−0.08 [−0.17, −0.02]	−0.14	−2.44^*^	0.012
*ΔR^2^*			0.009				0.018^**^				0.011^*^	
Total *R^2^*			0.076				0.060				0.071	

*p < 0.05, **p < 0.01, ***p < 0.001. B refers to the unstandardized coefficients and *β* refers to the standardized coefficients. Pedagogies in cultural education were binary variables (high/low frequency) and mean-centred. VIFs < 1.1 indicate no multicollinearity.

### Simple slope analyses

**Figure 1:**
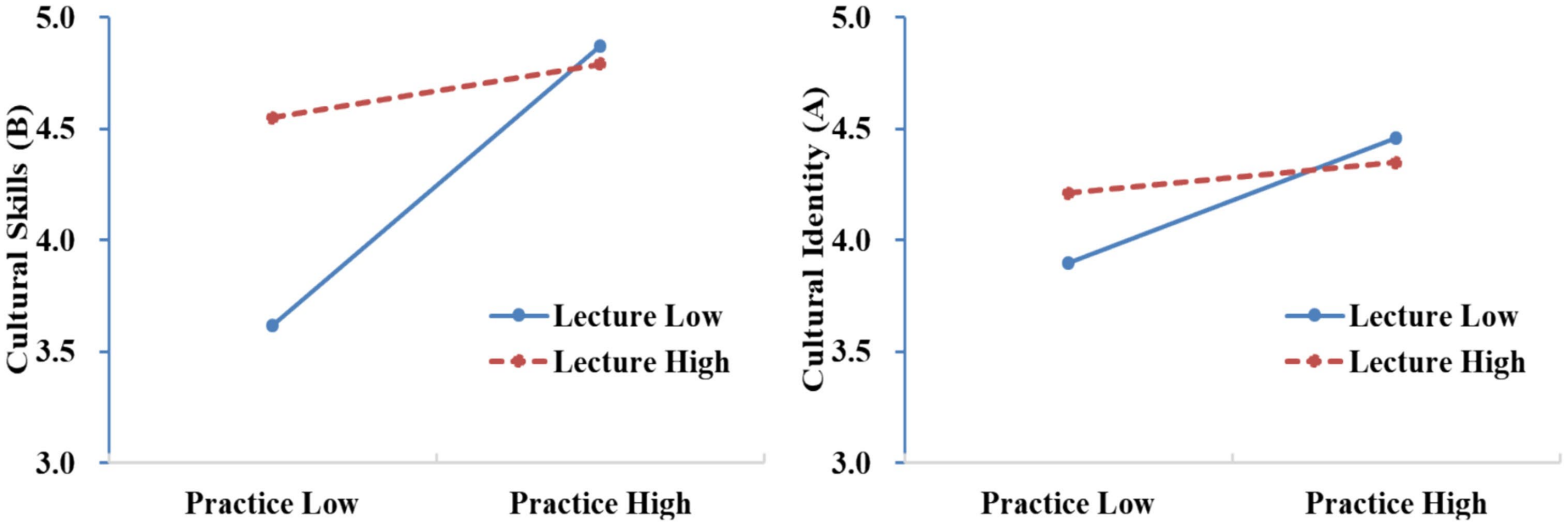
Simple slope plots for cultural skills and cultural identity.

To further explore the significant Lecture × Practice interaction for cultural skills and identity, simple slope analyses were conducted using PROCESS Model 1 (see Figure 1). The effect of practice-based pedagogy on cultural skills was significant in the low-lecture group (*β* = 1.25, *p* < 0.001). In the high-lecture group, this effect was substantially weakened and no longer significant (*β* = 0.24, *p* = 0.11). For cultural identity, a similar pattern was observed. Practice-based pedagogy had a significant positive effect in the low-lecture group (*β* = 0.56, *p* = 0.002). However, in the high-lecture group, this effect was reduced to marginal significance (*β* = 0.14, *p* = 0.072).

### Sensitivity analyses

To examine the robustness of the results, sensitivity analyses using complete-case data (*N* = 315) were conducted. Both the hierarchical regressions and the simple slope analyses showed a consistent pattern of findings (see Supplementary Material). Specifically, the Lecture × Practice interaction remained negatively significant for cultural skills and identity, and the simple slopes showed the same decreasing trend from low to high lecture conditions, supporting the robustness of the saturation effect.

## Discussion

This study examined the effectiveness of formal cultural education in enhancing the acculturation of international students in Chinese universities. Guided by Ward’s ABC framework, our findings both corroborate and extend prior research on acculturation. Overall, cultural education was associated with enhanced cognitive (C), behavioral (B), and affective (A) adaptation among international students, supporting the integrative perspective of the ABC model. While prior meta-analyses have shown that cross-cultural training is generally effective (Powell et al., 2024), the present study reveals a critical boundary condition that such benefits diminish when pedagogies are combined at high frequency in Confucian classrooms. Specifically, when both lecture-based and practice-based pedagogies were used frequently, the combination negatively affected students’ cultural skills (B) and identity (A). This result points to a potential saturation effect specific to the Confucian classroom context and challenges the universal “more-is-better” assumption.

The positive main effects align with the goals of cultural education policy in China, which encourages student participation in activities related to China’s national conditions and social interactions (Ministry of Education of the People’s Republic of China (Ministry of Education of the People's Republic of China (MEPRC), 2018). Through such engagement, students encounter authentic cultural contexts, which facilitate cognitive integration and likely lead to gains in knowledge, skills, and identity, consistent with previous studies (e.g., Bai et al., 2023; Wang et al., 2021a, 2021b). Our hierarchical regression design, which isolated the net contribution of each pedagogy while accounting for the other, mirrors the real-world conditions of Chinese cultural education, where both methods coexist. The analysis revealed that practice-based teaching uniquely enhanced all ABC dimensions, suggesting its foundational role. Lecture-based pedagogy showed a selective, unique benefit for skill development (B), possibly indicating that it requires the scaffolding provided by practice to effectively transfer knowledge (C) or foster identity (A).

The negative interaction effect, where the high-frequency combination of pedagogies reduced behavioral and affective adaptation, is the most significant finding. It suggests a cultural threshold that the benefits of practice diminish when lectures are used intensively in Confucian classrooms. We argue that this saturation effect may be explained by the cognitive load theory (Sweller, 1988; Sweller et al., 2019). According to cognitive load theory, working memory has a limited capacity, and ineffective instructional design can overload it with extraneous processing. In Confucian classrooms, which are characterized by high power distance and high-context communication (Hofstede, 2011), students must dedicate significant cognitive resources to decoding implicit teacher cues and navigating hierarchical norms. The frequent switching between pedagogical modes then may add substantial extraneous cognitive load, a phenomenon similar to that found in complex learning contexts (Seufert, 2018). When the total cognitive load exceeds working memory capacity, resources for deep processing are compromised, particularly affecting the more integrative behavioral and affective domains. Cognitive gains (C) may be more resilient to this overload because knowledge acquisition relies less on sustained emotional and behavioral engagement. Thus, the saturation effect aligns with the unique cognitive demands of Confucian-influenced learning environments.

### Theoretical and practical implications

Theoretically, this study extends Ward’s ABC framework by identifying a critical boundary condition (i.e., saturation effect) for blended pedagogy, thereby challenging the Western “more-is-better” paradigm in pedagogical design. Specifically, it demonstrates that the effectiveness of blended pedagogy is not universal but bounded by cultural contexts and pedagogical intensity. This effect underscores the importance of subjective appraisals in acculturation (Ward, 1996), particularly in high-context cultures where learning is deeply ritualized (Li, 2012). It introduces necessary cultural and contextual boundaries to the framework (Berry, 2005), affirming that pathways to successful adaptation are not universal but are moderated by the host institution’s cultural environment. Thus, the study shows that “more” is not always “better” in cross-cultural education.

In practice, the findings provide clear guidance for designing cultural education programs to support international students’ adaptation in China and similar contexts. To mitigate the cognitive overload identified above, programs should be strategically blended rather than formally blended. Based on our data, we suggest that in similar contexts, practice-based activities should form the core of instructional design, judiciously supplemented by theoretical lectures. For instance, adopting a “practice-first, lecture-later” sequence or explicitly increasing the proportion of practice-based instruction in the curriculum (e.g., to approximately 70%) could help mitigate cognitive overload and optimize student adaptation outcomes. This is particularly relevant for Confucian-heritage classrooms, where pedagogical intensity needs to be strategically calibrated rather than maximized.

### Limitations and future directions

The present study sheds light on the effectiveness of cultural education in China for international students. However, the study has several limitations. First, while representative of current demographics, the sample was limited to international students from Asia and Africa studying in China. Future research should test whether the saturation effect persists with students from more culturally distant regions. Second, the cross-sectional design precludes causal inferences. Longitudinal studies are needed to establish the directionality of the observed effects. Third, pedagogy frequency was self-reported and measured as binary, based on students’ subjective perception without a standardized definition. This results in information loss compared to continuous measures. Future research should employ Likert-type frequency scales. Finally, although we propose cognitive load as the underlying mechanism, it was not measured directly. Future studies should incorporate direct measures of cognitive load to empirically test this proposed mechanism. Replications in individualistic cultures are also necessary to test the effect’s cultural boundary conditions.

## Conclusion

This study examined whether blended pedagogy of combining lectures and practice affects international students’ acculturation in Confucian classrooms. Analysis of survey data revealed several main findings. First, practice-based pedagogy positively contributed to all ABC domains (cognitive, behavioral, and affective). Second, lecture-based pedagogy alone only benefited behavioral skills. Third, and most critically, the high-frequency combination of the two pedagogies produced a saturation effect. It significantly reduced students’ behavioral adaptation and cultural identity, although cognitive knowledge remained unaffected. These findings challenge the implicit “more-is-better” assumption in cross-cultural education and suggest that pedagogical intensity must be strategically calibrated. We conclude that in Confucian-heritage classrooms, a practice-oriented approach with judicious use of lectures may be more effective than intensive blending.

## Data Availability

The raw data supporting the conclusions of this article will be made available by the authors, without undue reservation.
