# Supplementary material for: The saturation effect of blended pedagogy on international students’ acculturation in Confucian classrooms
Source: Front Psychol. 2026 Jun 12;17:1803766. doi: 10.3389/fpsyg.2026.1803766 (PMC13351086; doi:10.3389/fpsyg.2026.1803766)
Supplement: Supplementary file 1 [file Supplementary_file_1.pdf]

Appendix

Table S1. Hierarchical regression results (*N* = 315)

Predictor variables	Cultural knowledge (C)				Cultural skills (B)				Cultural identity (A)			
	B [95% CI]	*β*	*t*	*sr²*	*B* [95% CI]	*β*	*t*	*sr²*	*B* [95% CI]	*β*	*t*	*sr²*
Demographics												
Gender	-0.07 [-0.39, 0.25]	-0.02	-0.42	0.001	0.03 [-0.30, 0.36]	0.01	0.18	<0.001	-0.11 [-0.28, 0.06]	-0.07	-1.25	0.005
Degree level	<0.01 [-0.17, 0.17]	<0.01	0.01	<0.001	-0.05 [-0.23, 0.13]	-0.03	-0.57	0.001	0.03 [-0.07, 0.12]	0.03	0.56	0.001
Religion	0.07 [-0.43, 0.56]	0.02	0.27	<0.001	-0.08 [-0.59, 0.43]	-0.02	-0.31	<0.001	-0.10 [-0.37, 0.16]	-0.04	-0.77	0.002
Duration of stay	0.09 [0.00, 0.17]	0.11	1.99*	0.013	0.03 [-0.06, 0.12]	0.04	0.62	0.001	0.05 [0.00, 0.09]	0.11	1.92	0.012
Cultural distance	0.03 [-0.08, 0.14]	0.03	0.48	0.001	0.04 [-0.07, 0.16]	0.04	0.73	0.002	0.03 [-0.03, 0.09]	0.05	0.86	0.002
ΔR^2			0.015				0.007				0.023	
Cultural education												
Lecture-based	0.08 [-0.06, 0.22]	0.06	1.08	0.004	0.16 [0.02, 0.31]	0.12	2.20*	0.016	0.03 [-0.05, 0.10]	0.04	0.73	0.002
Practice-based	0.33 [0.18, 0.47]	0.24	4.43***	0.060	0.24 [0.09, 0.39]	0.18	3.19**	0.032	0.12 [0.04, 0.20]	0.17	3.08**	0.030
ΔR^2			0.065***				0.053***				0.033**	
Interaction												
Lecture×Practice	-0.11[-0.25, 0.04]	-0.08	-1.45	0.006	-0.17 [-0.32, -0.02]	-0.13	-2.28*	0.017	-0.10 [-0.17, -0.02]	-0.14	-2.44*	0.019
ΔR^2			0.006				0.016*				0.018*	
Total R^2			0.086				0.076				0.074	

Note. *$p < 0.05$, **$p < 0.01$, ***$p < 0.001$. *B* refers to the unstandardized coefficients, and *β* refers to the standardized coefficients. Pedagogies in cultural education were binary variables (high/low frequency) and mean-centred. VIFs < 1.1 indicate no multicollinearity.

Table S2. Simple slope analysis results (*N* = 315)

Dependent Variable	Lecture Group	*β*	*SE*	*t*	*p*	95% CI
Cultural skills (B)	Low	1.30	0.39	3.36	< 0.001	[0.54, 2.06]
	High	0.34	0.17	2.06	0.040	[0.02, 0.67]
Cultural identity (A)	Low	0.70	0.20	3.46	< 0.001	[0.30, 1.10]
	High	0.16	0.09	1.89	0.059	[-0.01, 0.34]

Note. β refers to the standardized coefficients.
